# Altered functional connectivity architecture of the brain in medication overuse headache using resting state fMRI

**DOI:** 10.1186/s10194-017-0735-0

**Published:** 2017-02-20

**Authors:** Zhiye Chen, Xiaoyan Chen, Mengqi Liu, Zhao Dong, Lin Ma, Shengyuan Yu

**Affiliations:** 1 Department of Radiology, Chinese PLA General Hospital, Beijing, 100853 China; 2 Department of Neurology, Chinese PLA General Hospital, Beijing, 100853 China; 3 Department of Radiology, Hainan Branch of Chinese PLA General Hospital, Beijing, 100853 China

**Keywords:** Medication overuse headache, Functional connectivity density, Functional connectivity, Brain, Magnetic resonance imaging

## Abstract

**Background:**

Functional connectivity density (FCD) could identify the abnormal intrinsic and spontaneous activity over the whole brain, and a seed-based resting-state functional connectivity (RSFC) could further reveal the altered functional network with the identified brain regions. This may be an effective assessment strategy for headache research. This study is to investigate the RSFC architecture changes of the brain in the patients with medication overuse headache (MOH) using FCD and RSFC methods.

**Methods:**

3D structure images and resting-state functional MRI data were obtained from 37 MOH patients, 18 episodic migraine (EM) patients and 32 normal controls (NCs). FCD was calculated to detect the brain regions with abnormal functional activity over the whole brain, and the seed-based RSFC was performed to explore the functional network changes in MOH and EM.

**Results:**

The decreased FCD located in right parahippocampal gyrus, and the increased FCD located in left inferior parietal gyrus and right supramarginal gyrus in MOH compared with NC, and in right caudate and left insula in MOH compared with EM. RSFC revealed that decreased functional connectivity of the brain regions with decreased FCD anchored in the right dorsal-lateral prefrontal cortex, right frontopolar cortex in MOH, and in left temporopolar cortex and bilateral visual cortices in EM compared with NC, and in frontal-temporal-parietal pattern in MOH compared with EM.

**Conclusions:**

These results provided evidence that MOH and EM suffered from altered intrinsic functional connectivity architecture, and the current study presented a new perspective for understanding the neuromechanism of MOH and EM pathogenesis.

## Background

Medication-overuse headache (MOH) is a secondary form of chronic headache deriving from episodic migraine (EM) related to the overuse of triptans, analgesics and other acute headache medications [1–3]. Previous studies demonstrated that functional and structural changes were identified in MOH [2, 4–6], however, functional connectivity density (FCD) over the whole brain and functional connectivity with seed-based FCD were not still performed in MOH.

A previous study presented no significant change in morphometry using voxel-based morphometry (VBM) [2], however, Riederer et al. recognized that increased gray matter in the midbrain presented in MOH [5]. Further functional connectivity using resting-state functional MRI (rs-fMRI) demonstrated that altered functional connectivity was revealed in MOH, and suggested that MOH is associated with intrinsic brain network changes rather with macrostructural changes [2]. Therefore, some studies also demonstrated that MOH patients were characterized by an altered nucleus accumbens functional connectivity of motivational circuits [1], and abnormal connectivity between the PAG and other pain modulatory (frontal) regions in MOH were consistent with dysfunctional central pain control recently [7]. The previous study also confirmed that the functional connectivity of marginal division of neostriatum increased in MOH compared with EM [4]. Make a general survey of functional connectivity, the selected seed points analysis was based on the documents with some priori knowledge and constrained the degree of functional connectivity changes on the priori selection of specific seed regions, which would generate subjective bias in evaluating the intrinsic functional changes over the whole brain. Therefore, it was very important to define the seed points of functional connectivity to understand the pathophysiology in MOH.

FCD was a voxel-wise data-driven method to calculate the functional connectivity over the whole brain and would overcome the limitations of seed-based approaches for the identification of functional hub in the human brain [8, 9], although seed-based approaches did so to test very specific hypotheses in migraine [4, 10, 11] while not totally detected the other unreported brain regions with alter functional hub in migraine. FCD had been used to identify the abnormal functional connectivity in children with attention-deficit/hyperactivity disorder [12], gender differences in cognitive style and behaviors and in the prevalence of neuropsychiatric diseases [13], Balance in Young Patients with Traumatic Axonal Injury [14], Parkinson’s disease [15], and pain-related brain regions of female migraine patients without aura [16]. However, FCD over the whole brain was not applied to detect the abnormal functional hub on the brain in the MOH patients.

In this study, we would hypothesize that disrupted functional connectivity of the brain presented in MOH patients. To address the hypothesis, FCD was performed to identify the brain regions with abnormal functional hubs over the whole brain in MOH, episodic migraine (EM) and normal controls (NC). Secondly, the cluster with abnormal FCD was considered as a specific seed point to evaluate the altered degree of functional connectivity in MOH.

## Methods

### Subjects

Thirty-seven MOH patients and 18 episodic migraine (EM) patients were recruited from International Headache Center, Department of Neurology, Chinese PLA General Hospital, and inclusion criteria was based on the International Classification of Headache Disorders, third Edition (beta version) (ICHD −3 beta) [17]. All the patients underwent Visual Analogue Scale (VAS) for the pain intensity evaluation, Migraine Disability Assessment questionnaire (MIDAS), Hamilton Anxiety Scale (HAMA) for the anxiety evaluation, Hamilton Depression Scale (HAMD) for the depression evaluation and Montreal Cognitive Assessment (MoCA) for the cognitive function evaluation. The exclusion criteria were listed as following: cranium trauma, central nervous illness such as cerebral infarction, malacia, brain tumor, and metabolic disorders etc., psychotic disorder, and regular use of a psychoactive or hormone medication. 32 normal controls (NCs) were recruited form hospital staffs and their relatives. All the subjects received general physical examination and neurological examination and were normotensive (≤140/90 mmHg), and free from cardiovascular, metabolic and psychiatric disorders. All the subjects were right-handed and underwent MRI conventional examination to exclude the subjects with cerebral infarction, malacia or brain tumors etc. The alcohol, nicotine, caffeine and other substances were avoided at least 12 h before MRI examination. MRI scans were taken in the interictal stage at least three days after a migraine attack for MOH and EM patients. Written informed consent was obtained from all participants according to the approval of the ethics committee of the Chinese PLA General Hospital.

### MRI acquisition

Images were acquired on a GE 3.0 T MR system (DISCOVERY MR750, GE Healthcare, Milwaukee, WI, USA) and a conventional eight channel quadrature head coil was used. All the subjects were instructed to lie in a supine position, and form padding was used to limit head movement. A pulse oximeter and respiratory belt were worn to monitor cardiac and respiratory signal during the resting-state fMRI data acquisition. Conventional T2-weighted images were obtained first. Then a high resolution three-dimensional T1-weighted fast spoiled gradient recalled echo (3D T1-FSPGR) sequence was performed, which generated 360 contiguous axial slices [TR (repetition time) = 6.9 ms, TE (echo time) = 3.0 ms, flip angle = 15°, FOV (field of view) = 25.6 cm × 25.6 cm, Matrix = 256 × 256, slice thickness = 1 mm]. Lastly, the resting-state fMRI was performed, where subjects were instructed to relax, keep their eyes closed, stay awake, remain still, and clear their heads of all thoughts. Functional images were obtained by using a gradient echo-planar imaging (EPI) sequence (TR = 2000 ms, TE = 30 ms, flip angle = 90, slice thickness = 4 mm, slice gap = 1 mm, FOV = 24 cm × 24 cm, Matrix = 64 × 64), and 180 continuous EPI functional volumes were acquired axially over 6 min. All the subjects did not complain any discomfort and feel asleep during scanning. No obvious structural damage was observed based on the conventional MR images.

### Data processing

MR resting-state functional images were processed using Statistical Parametric Mapping 8 (SPM8) (http://www.fil.ion.ucl.ac.uk/spm), DPABI (a toolbox for Data Processing & Analysis of Brain Imaging)(V2.1_160415) [18] and resting-state fMRI data analysis toolkit (REST v1.8) [19] running under MATLAB 7.6 (The Mathworks, Natick, MA, USA).

The data preprocessing was carried out as following: (1) The first ten volumes of each functional time course was discarded to allow for T1 equilibrium and the participants to adapt; (2) Slice timing; (3) Head motion correction; (4) Spatial normalization. These steps were performed by SPM8. No subjects had head motion with more than 1.5 mm displacement in X, Y, and Z direction or 1.50 of any angular motion throughout the course of the scanning. The physiological noise including cardiac and respiratory signals were regressed out from the functional data as other covariates. The linear trend removal and temporal band-pass filtering (0.01-0.08 Hz) was performed by REST [19].

The FCD analysis were performed as following [20]: (1) Voxel-based whole brain correlation analysis was performed to compute the whole-brain connectivity for the each voxel within the gray matter mask (r > 0.25) [21–24]; (2) The sum of the weights of the significant connections was obtained for each voxel and considered as FCD; (3) The individual FCD was converted into a z-score map and was analyzed using two sample *t*-test.

The resting-state functional connectivity (RSFC) analysis was performed as following: (1) Spatial smooth (full width at half maximum (FWHM) = 6 mm) using SPM8; (2) The seed regions were obtained from the binary cluster masks based on the results of the FCD analysis; (3) Functional connectivity computation of the seed regions were performed using REST(v1.8). The time course of the seed regions were extracted, and Pearson correlation were used to calculated functional connectivity between the extracted time course and the averaged time courses of the whole brain in a voxel-wise manner. The white matter, CSF, and the six head motion parameters were used as covariates [19]. (4) The individual r-maps were normalized to Z-maps using Fisher’s R-to-Z transformation. (5) The abnormal clusters based on the statistical parametric mapping were generated binary mask, and the connectivity strength of the altered brain region was extracted based on the Z-maps.

### Statistical analysis

The age, HAMA, HAMD and MoCA were performed with analysis of variance (ANOVA). The diseased duration, VAS, and MIDAS were performed with two-sample *t* test. The gender was performed with Pearson Chi-Square text. These statistics was processed using IBM SPSS 19.0, and the *P* value of less than 0.05 was considered to indicate a statistically significant difference.

FCD analysis and RSFC analysis were performed with two-sample *t.* test based SPM8 software with age and gender as covariants. Significance was set at a *P* value of < 0.05 with false discovery rate (FDR) correction. The minimal number of contiguous voxels was based on the expected cluster size. The statistical maps were masked on SPM8 T1 template.

Partial correlation analysis was applied to the connectivity strength of the positive brain region with control for age and gender in each compared groups. The *P* value of less than 0.05 was considered to indicate a statistically significant difference.

## Results

### Demography and neuropsychological test

Demography and neuropsychological scores were shown in Table 1. Age and gender showed no significant difference among each group (*P* > 0.05). HAMA and HAMD score showed higher in MOH than that in EM and NC, and MoCA score presented lower in MOH than that in EM and NC (*P* < 0.05). The diseased duration and MIDAS score presented higher in MOH than that in EM (*P* < 0.05), and VAS score showed no significant difference between MOH and EM (*P* > 0.05). The type of overused medication in MOH patients included:simple analgesics (34/37, simple triptans (1/37), and combination analgesics (2/37).

**Table 1:** The clinical characteristics of MOH patients and NCs

	MOH	EM	NC	F(T) value	*P* value
Num(F/M)	37(30/7)	18(14/4)	32(20/12)		
Age	41.27 ± 9.27	33.39 ± 10.69	41.34 ± 10.89	4.13	0.02
HAMA*	18.35 ± 8.65	15.67 ± 9.85	10.19 ± 2.98	10.56	0.00
HAMD*	20.03 ± 12.68	10.89 ± 7.26	8.03 ± 4.34	17.40	0.00
MoCA*	23.08 ± 3.80	29.17 ± 1.47	27.16 ± 2.32	31.22	0.00
DD(yrs)*	18.57 ± 9..08	12.44 ± 8.07	NA	2.00	0.02
VAS	8.16 ± 1.57	8.33 ± 1.50	NA	2.00	0.70
MIDAS*	124.43 ± 72.62	16 ± 17.94	NA	2.00	0.00

*There was significant difference among each group (*P* < 0.05)

*MOH* medication overuse headache, *NC* normal control, *HAMA* Hamilton Anxiety Scale, *HAMD* Hamilton Depression Scale, *MoCA* Montreal Cognitive Assessment, *DD* disease duration, *VAS* Visual Analogue Scale, *MIDAS* Migraine Disability Assessment questionnaire, *NA* not available

### FCD analysis over the whole brain among MOH, EM and NC

**Fig. 1:**
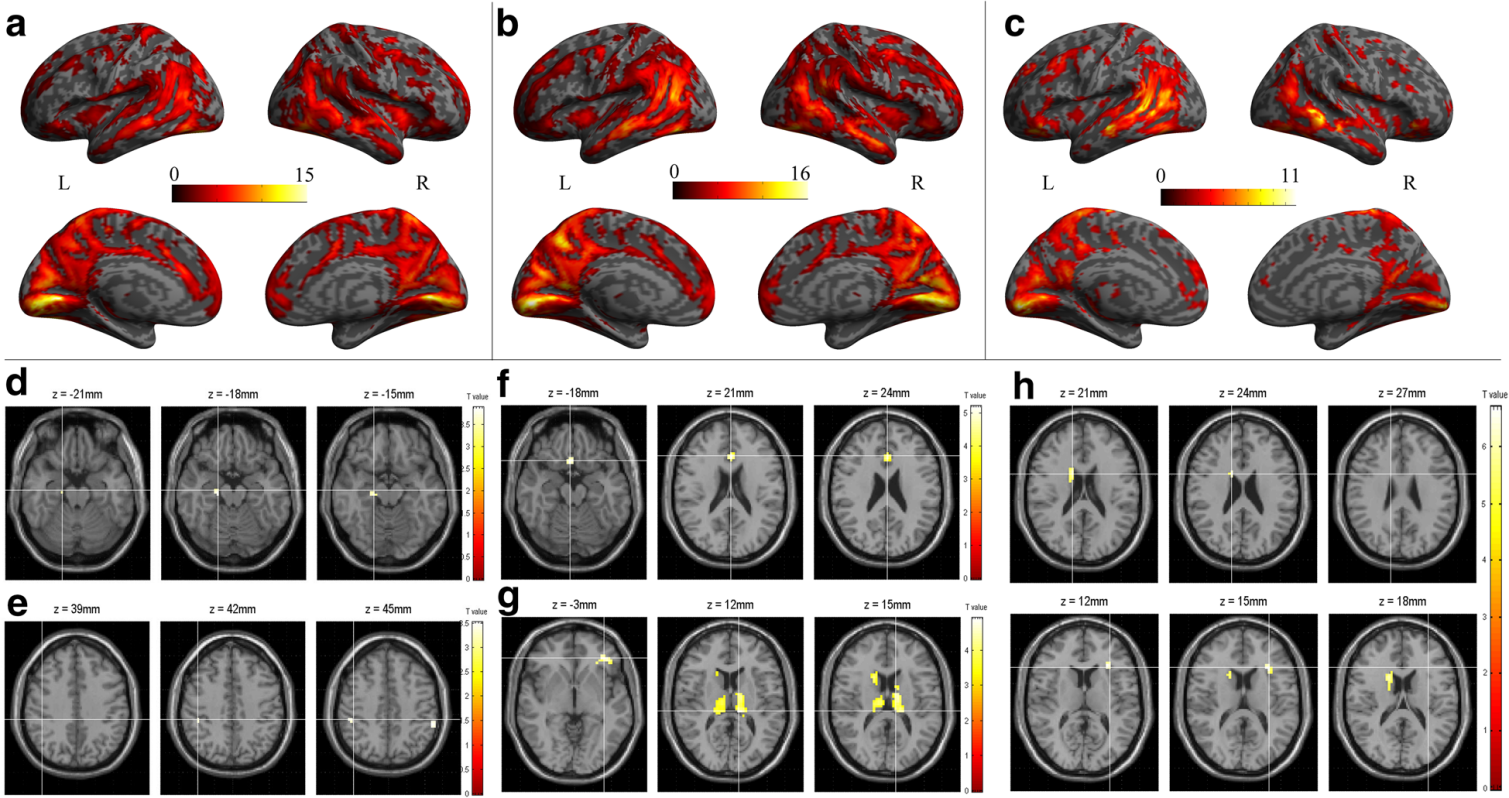
**a**, Fucntioal connectivity density (FCD) distribution in normal controls; **b**, FCD distribution in MOH patients; **c**, FCD distribution in EM patients; **d**, The brain regions with significant decreased FCD in MOH compared with NC; **e**, The brain regions with significant increased FCD in MOH compared with NC; **f**, The brain regions with significant decreased FCD in EM compared with NC; **g**, The brain regions with significant increased FCD in EM compared with NC; **h**, The brain regions with significant decreased FCD in MOH compared with EM. L, left hemisphere; R, right hemisphere

For the NCs, FCD was spatially distributed in calcarine cortex, cuneus, precuneus, midde cingulate cortex (MCC) and posterior cingulate cortex (PCC), anterior cingulate cortex (ACC), medial prefrontal cortex (mPFC), lateral prefrontal cortex(lPFC), inferior parietal cortex and temporal lobe (Fig. 1a). The spatial distribution of FCD was similar in MOH and EM with that in NC, and some regions shows more clusters and had higher significance in MOH (Fig. 1b) and EM (Fig. 1c).

Table 2 showed that the brain region with decreased FCD located in right parahippocampal gyrus (PHG) (Fig. 1d), and the increased FCD located in left inferior parietal gyrus and right supramarginal gyrus in MOH patients compared with NCs(Fig. 1e). The decreased FCD of EM located in left anterior cingulate cortex and right inferior orbital frontal gyrus (Fig. 1f), and the increased FCD of EM located in bilateral thalamus, right caudate and left inferior orbital frontal gyrus compared with NC (Fig. 1g). MOH patients had decreased FCD in right caudate and left insula (Fig. 1h), and showed no increased FCD compared with EM.

**Table 2:** The brain regions with altered functional connectivity density over the whole brain among MOH, EM and NC

	Anatomic region	MNI-space			Cluster size	Peak T value
		X	Y	Z		
MOH < NC						
	ParaHippocampal_R	21	−18	−18	12	3.88
MOH > NC						
	Parietal_Inf_L	−54	−39	48	20	3.98
	SupraMarginal_R	51	−33	48	10	3.52
EM < NC						
	Cingulum_Ant_L	0	27	21	32	5.2
	Frontal_Inf_Orb_R	15	15	−24	25	3.95
EM > NC						
	Thalamus_L	−9	−27	12	112	4.91
	Caudate_R	15	9	21	73	4.79
	Thalamus_R	12	−27	9	91	4.47
	Frontal_Inf_Orb_L	−36	39	−3	62	4.34
MOH < EM						
	Caudate_R	15	6	24	34	6.82
	Insula_L	−30	24	15	19	6.68

*MNI* Montreal Neurological Institute, *X*, *Y*, *Z* coordinates of the primary maximum of the cluster, *MOH < NC* decreased FCD in MOH compared with NC, *MOH > NC* increased FCD in MOH compared with NC, *EM < NC* decreased FCD in EM compared with NC, *EM > NC* increased FCD in EM compared with NC, *MOH < EM* decreased FCD in MOH compared with EM

### RSFC analysis between MOH and NC

Based on the FCD analysis results, right papahippocampal gyrus showed decreased FCD, and it was set as seed region to compute RSFC to identify the altered resting-state functional architecture in MOH compared with NC.

**Fig. 2:**
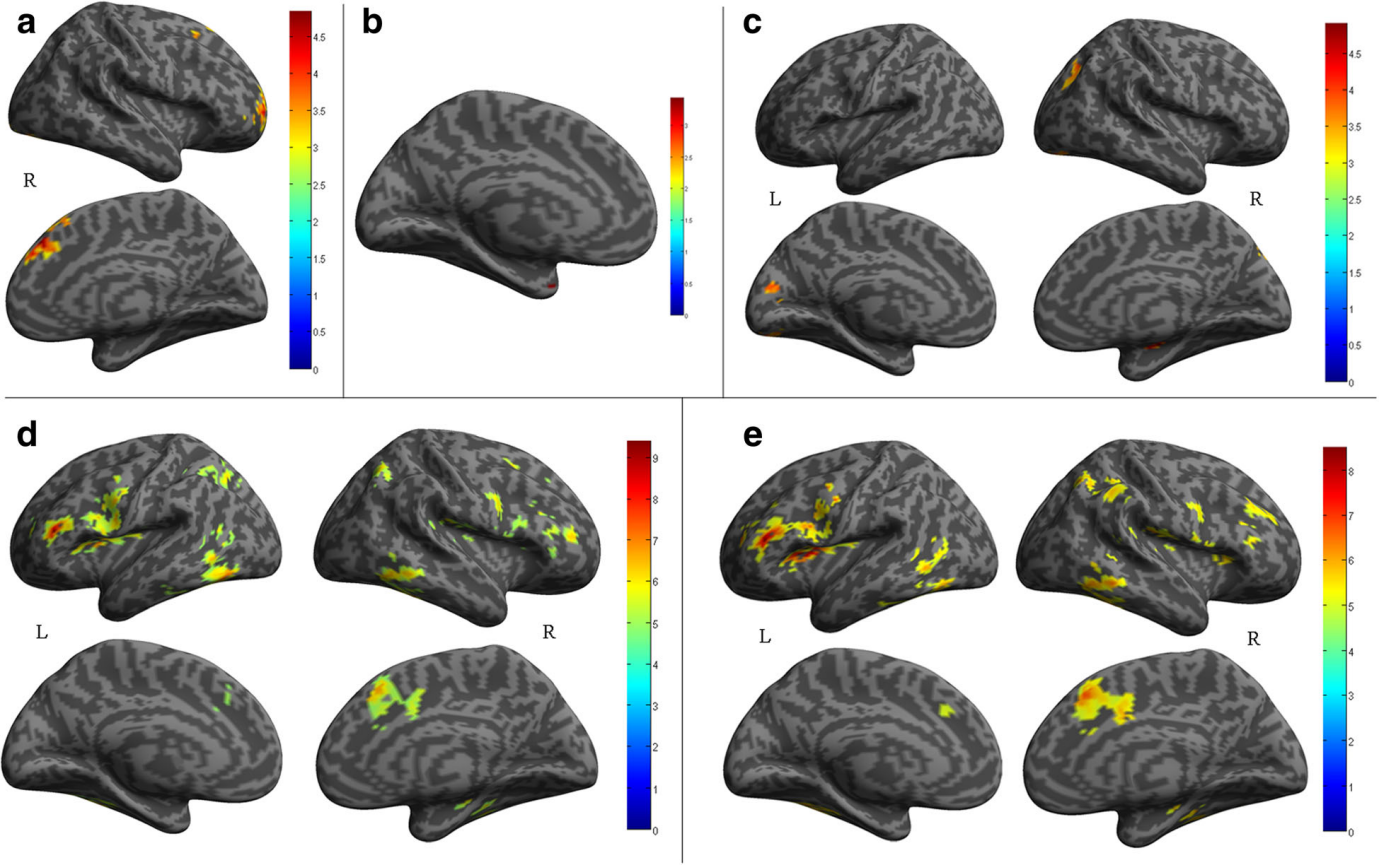
**a**, The decreased RSFC of the right parahippocampal gyrus in MOH compared with NC; **b**, The decreased RSFC of the left ACC in EM compared with NC; **c**, The decreased RSFC of the right IFG-orb in EM compared with NC; **d**, The decreased RSFC of the right caudate in MOH compared with EM; **e**, The decreased RSFC of the left insula in MOH compared with EM; L, left hemisphere; R, right hemisphere

Table 3 showed that the decreased RSFC of right parahippocampal gyrus located in right medial superior frontal gyrus (BA9, dorsolateral prefrontal cortex, dlPFC) and superior frontal gyrus (BA10, frontopolar cortex) (Fig. 2). There was no significant correlation between the connectivity strength of dlPFC and frontopolar cortex and the clinical variables (disease duration, VAS, MIDAS, HAMA, HAMD and MoCA). There was no increased RSFC of right parahippocampal gyrus in MOH patients compared with NC.

**Table 3:** The decreased functional connectivity of abnormal brain region with altered FCD among NC, EM and MOH

Seed region		Anatomic region	MNI-space			Cluster size	T value
			X	Y	Z		
MOH vs NC							
ParaHippocampal_R	MOH < NC	Frontal_Sup_Medial_R	6	42	39	199	4.47
		Frontal_Sup_R	27	63	9	140	4.39
EM vs NC							
Cingulum_Ant_L							
	EM < NC	Temporal_Pole_Sup_L	−27	9	−30	24	4.72
Frontal_Inf_Orb_R							
	EM < NC	Hippocampus_R	30	−18	−12	31	4.92
		Calcarine_L	−9	−78	15	45	4.32
		Occipital_Sup_R	27	−69	36	91	4.06
		Lingual_L	−18	−87	−15	43	3.89
MOH vs EM							
Caudate_R							
	MOH < EM	Frontal_Inf_Tri_L	−33	30	12	715	8.87
		Temporal_Sup_R	45	−24	15	133	6.98
		Temporal_Mid_R	66	−54	−6	337	6.85
		Frontal_Sup_Medial_R	9	27	45	307	6.78
		Fusiform_L	−45	−48	−21	276	6.47
		Angular_R	33	−57	45	232	6.28
		Insula_R	33	24	9	198	6.00
		Rolandic_Oper_R	63	12	12	137	5.99
Insula_L							
	MOH < EM	Frontal_Inf_Tri_L	−33	30	12	677	9.48
		Occipital_Inf_L	−48	−66	−15	352	7.38
		Temporal_Sup_R	45	−27	15	110	7.04
		Frontal_Sup_Medial_R	12	27	45	253	6.90
		Temporal_Inf_R	57	−51	−9	275	6.83
		Frontal_Mid_R	45	45	9	132	6.63
		Parietal_Sup_R	36	−60	54	138	6.39
		Occipital_Mid_L	−27	−63	42	103	6.25
		Frontal_Inf_Oper_R	51	9	30	182	6.24

*dmPFC* dorsal-medial prefrontal cortex, *vlPFC* ventral-lateral prefrontal cortex, *MNI* Montreal Neurological Institute, *X*, *Y*, *Z* coordinates of the primary maximum of the cluster

The brain regions with increased FCD were left inferior parietal gyrus and right supramarginal gyrus in MOH compared with NC, which were also set as seed regions to compute RSFC maps. However, there was significant difference for the RSFC maps between MOH and NC.

### RSFC analysis between EM and NC

The brain regions with decreased FCD located in left anterior cingulate cortex (ACC) and right inferior orbitofrontal gyrus (OFC) in EM compared with NC. RSFC analysis showed that there was a decreased FC with left superior temporal pole (BA28) for left ACC and a decreased FC with right hippocampus, left calcarine gyrus(BA17, primary visual cortex), right superior occipital gyrus (BA19, visual association cortex) and lingual gyrus (BA18, visual association cortex) for right OFC in EM compared with NC (Table 3). There was no increased FC for left ACC and right OFC in EM compared with NC.

There was no significant correlation between the connectivity strength of the positive brain regions for left ACC and right OFC and the clinical variables in EM patients.

The brain regions with increased FCD in EM showed no altered RSFC between EM and NC.

### RSFC analysis between MOH and EM

The brain regions with decreased FCD located in right caudate and left insula in MOH compared with EM. Table 3 showed that there was a decreased FC with left triangular part of inferior frontal gyrus (IFG_tri), right superior temporal gyrus, right middle temporal gyrus, right medial superior frontal gyrus, left fusiform gyrus, right angular gyrus, right insula and right Rolandic operculum for right caudate in MOH compared with EM. The left insula had a decreased FC with left IFG-tri, left inferior occipital gyrus, right superior temporal gyrus, right medial superior frontal gyrus, right inferior temporal gyrus, right middle frontal gyrus, right superior parietal gyrus, left middle occipital gyrus and right operculum part of inferior frontal gyrus (IFG-oper) in MOH compared with EM. These two brain regions showed no increased FC over the whole brain in MOH compared with EM.

There was no significant correlation between the connectivity strength of the positive brain regions for right caudate and left insula and the clinical variables in MOH patients.

## Discussion

The present study demonstrated altered local functional connectivity density (lFCD) in the brain of NC, MOH and EM patients, which had a powerful scaling for the numbers of functional connections, and was the main characteristic of the “scale-free” networks. In this study, the decreased FCD of right parahippocampal gyrus in MOH may be associated with the medication overuse because of the higher VAS score. The previous documents identified the decreased volume [25] and prolonged T2 relaxation times [26] in right parahippocampal gyrus in smoker and alcohol-use disorders, which suggested right parahippocampal might participate the dependence related processing. This study opportunely provided a clue for the dependence-related processing about the altered FCD in right parahippocampal gyrus in MOH patients. The MOH patients also presented lower MoCA score, and this cognitive impairment indicated that the decreased FCD of right parahippocampal gyrus was related with the cognitive ability [27].

The inferior parietal lobule and supramarginal gyrus were the pain-related brain region in MOH patients after beginning withdrawal [28], and increased FCD in these regions may reflect the intrinsic hub brain regions dysfunction in MOH. Although the increased FCD did not represent the deleterious change or compensatory effect [29] associated with migraine, the current findings of FCD changes confirmed the importance of network-level brain alterations in migraine patients.

Further functional connectivity of the cluster with decreased FCD in MOH presented right medial superior frontal gyrus (BA9, dorsolateral prefrontal cortex, dlPFC) and right superior frontal gyrus (BA10, frontopolar cortex). The previous studies identified the hypometabolism in the prefrontal cortex of MOH patients [30], functional alteration in orbitofrontal region of prefrontal cortex [31], and increased activity in the ventromedial prefrontal cortex when compared with detoxified MOH patients [32]. However, decreased functional connectivity of dlPFC was confirmed in this study, which indicated dlPFC might participate in pain modulation in MOH. Frontopolar cortex was positively related with neuropathic pain [33], and this region was not reported in MOH and the other types of migraine. Therefore frontopolar cortex may be a newfound brain region involved in the pain modulation in MOH using FCD methods.

Functional connectivity of the clusters with increased FCD did not show altered functional connectivity in MOH compared with NC, which further indicated that the brain regions with increased FCD may be reversible in function in MOH. These finding suggested the brain regions with increased FCD may predict a transitional response for the drugs overuse in MOH. However, the precisely neuromechanism should be investigated in future.

In EM patients, decreased FCD was mainly located in left anterior cingulated cortex (ACC) and right inferior orbitofrontal cortex (OFC), which might be associated with hypometabolism and altered pain processing network in ACC and OFC in EM [34–36], and it might also be consistent with dysfunction of antinociceptive systems in EM. The increased FCD may further indicate that the altered lFCD of thalamus, caudate and left OFC may predict the reversible functional changes in EM because of the functional connectivity of seed based on the increased FCD was not revealed in EM, which was same as that in MOH patients.

The decreased functional connectivity of left ACC mainly located in left superior temporopolar cortex (BA28) in EM patients compared with NC. VBM study demonstrated the left temporopolar showed decreased gray matter density in EM [37], and another classification study recognized that the structural characteristics of temporopolar cortex were considered as one of classifiers [38]. In this study, the decreased functional connectivity between left ACC and left temporopolar cortex suggested that the dysfunction of this pain modulatory network presented in EM. The decreased functional connectivity between right OFC and bilateral visual cortex demonstrated the dysfunctional visual information processing ability in EM, and which might be the trigger point of EM pathogenesis [39, 40]. Hippocampus showed greater pain-induced activation [41] and volume reduction in EM [42], and the right OFC-hippocampus network dysfunction in this study further revealed the decreased antinociceptive systems in EM patient.

FCD analysis demonstrated that the decreased FCD mainly located in right caudate and left insula in MOH patients compared with EM patients, which might speculate that caudate and insula were involved in MOH evolving from EM. However, a previous study presented OFC involvement in MOH evolving from EM, which showed hypometabolic before withdrawal and showed a hypermetabolic after withdrawal [43]. Further functional connectivity analysis demonstrated that the decreased functional connectivity of right caudate and left insula both presented fronto-temporal-parietal distribution pattern. This decreased functional connectivity pattern indicated that right caudate and left insula played a key role in MOH evolving from EM, and the precise neuomechanism should further be elucidated such as using FDG-PET and perfusion weighted imaging to explore the metabolism or perfusion function.

Partial correlation analysis demonstrated that the connectivity strength of the brain regions with alter functional connectivity showed no significant correlation with the clinical variables in MOH and EM patients, which suggested that the altered functional connectivity of the seed based on the clusters with abnormal FCD over the whole brain reflected the intrinsic dysfunction changes in MOH and EM, and it may be not changed with clinical state. However, the further neuromechanism should be investigated to elucidate the reciprocity between disrupted functional connectivity changes and the neuropsychological variables in migraine patients in future.

The present study focused on the FCD (voxel-based whole brain functional connectivity) and FC (region-of-interest, also called as seed regions) analysis in migraine. However, there were several limitations in our study. Firstly, the sample size of EM was relative small, and it would be necessary to increase the sample size in the future study. Secondly, this study was a sectional observation, and longitudinal study should be performed to observe MOH evolving from EM.

## Conclusions

In conclusion, the altered intrinsic functional connectivity architecture was identified in MOH and EM patients, and this study also provide a new perspective for understanding the neuromechanism of MOH and EM pathogenesis.

## Abbreviations

CM: Chronic migraine
EM: Episodic migraine
FCD: Functional connectivity density
MOH: Medication overuse headache
NC: Normal control
RSFC: Resting-state functional connectivity

## References

[1] Torta DM, Costa T, Luda E, Barisone MG, Palmisano P, Duca S (2016). Nucleus accumbens functional connectivity discriminates medication-overuse headache. Neuroimage Clin.

[2] Chanraud S, Di Scala G, Dilharreguy B, Schoenen J, Allard M, Radat F (2014). Brain functional connectivity and morphology changes in medication-overuse headache: Clue for dependence-related processes?. Cephalalgia.

[3] Tepper SJ (2012). Medication-overuse headache. Continuum (Minneap Minn).

[4] Chen Z, Chen X, Liu M, Liu S, Shu S, Ma L, Yu S (2016). Altered functional connectivity of the marginal division in migraine: a resting-state fMRI study. J Headache Pain.

[5] Riederer F, Gantenbein AR, Marti M, Luechinger R, Kollias S, Sandor PS (2013). Decrease of gray matter volume in the midbrain is associated with treatment response in medication-overuse headache: possible influence of orbitofrontal cortex. J Neurosci.

[6] Riederer F, Marti M, Luechinger R, Lanzenberger R, von Meyenburg J, Gantenbein AR (2012). Grey matter changes associated with medication-overuse headache: correlations with disease related disability and anxiety. World J Biol Psychiatry.

[7] Michels L, Christidi F, Steiger VR, Sandor PS, Gantenbein AR, Landmann G et al (2016) Pain modulation is affected differently in medication-overuse headache and chronic myofascial pain - A multimodal MRIstudy. Cephalalgia. doi:10.1177/0333102416652625 10.1177/0333102416652625 27250235

[8] Tomasi D, Volkow ND (2011). Association between functional connectivity hubs and brain networks. Cereb Cortex.

[9] Tomasi D, Volkow ND (2010). Functional connectivity density mapping. Proc Natl Acad Sci U S A.

[10] Chen Z, Chen X, Liu M, Dong Z, Ma L, Yu S (2017). Altered functional connectivity of amygdala underlying the neuromechanism of migraine pathogenesis. J Headache Pain.

[11] Coppola G, Di Renzo A, Tinelli E, Lepre C, Di Lorenzo C, Di Lorenzo G (2016). Thalamo-cortical network activity between migraine attacks: Insights from MRI-based microstructural and functional resting-state network correlation analysis. J Headache Pain.

[12] Tomasi D, Volkow ND (2012). Abnormal functional connectivity in children with attention-deficit/hyperactivity disorder. Biol Psychiatry.

[13] Tomasi D, Volkow ND (2012). Gender differences in brain functional connectivity density. Hum Brain Mapp.

[14] Caeyenberghs K, Siugzdaite R, Drijkoningen D, Marinazzo D, Swinnen SP (2015). Functional connectivity density and balance in young patients with traumatic axonal injury. Brain Connect.

[15] Zhang J, Bi W, Zhang Y, Zhu M, Feng H, Wang J, Jiang T (2015). Abnormal functional connectivity density in Parkinson’s disease. Behav Brain Res.

[16] Gao Q, Xu F, Jiang C, Chen Z, Chen H, Liao H, Zhao L (2016). Decreased functional connectivity density in pain-related brain regions of female migraine patients without aura. Brain Res.

[17] Headache Classification Committee of the International Headache Society (IHS) (2013) The International Classification of Headache Disorders, 3rd edition (beta version). Cephalalgia. 33:629–808. 10.1177/0333102413485658 23771276

[18] Chao-Gan Y, Yu-Feng Z (2010). DPARSF: a MATLAB toolbox for “pipeline” data analysis of resting-state fMRI. Front Syst Neurosci.

[19] Song XW, Dong ZY, Long XY, Li SF, Zuo XN, Zhu CZ (2011). REST: a toolkit for resting-state functional magnetic resonance imaging data processing. PLoS ONE.

[20] Zhou Y, Wang Y, Rao LL, Liang ZY, Chen XP, Zheng D (2014). Disrutpted resting-state functional architecture of the brain after 45-day simulated microgravity. Front Behav Neurosci.

[21] Yan CG, Craddock RC, Zuo XN, Zang YF, Milham MP (2013). Standardizing the intrinsic brain: towards robust measurement of inter-individual variation in 1000 functional connectomes. Neuroimage.

[22] Weng Y, Qi R, Liu C, Ke J, Xu Q, Wang F et al (2016) Disrupted functional connectivity density in irritable bowel syndrome patients. Brain Imaging Behav. doi:10.1007/s11682-016-9653-z 10.1007/s11682-016-9653-z 27848148

[23] Baliki MN, Petre B, Torbey S, Herrmann KM, Huang L, Schnitzer TJ (2012). Corticostriatal functional connectivity predicts transition to chronic back pain. Nat Neurosci.

[24] Noble S, Scheinost D, Finn ES, Shen X, Papademetris X, McEwen SC et al (2016) Multisite reliability of MR-based functional connectivity. Neuroimage 146:959–70 10.1016/j.neuroimage.2016.10.020 PMC5322153 27746386

[25] Ding X, Yang Y, Stein EA, Ross TJ (2015). Multivariate classification of smokers and nonsmokers using SVM-RFE on structural MRI images. Hum Brain Mapp.

[26] Bagga D, Modi S, Poonia M, Kaur P, Bhattacharya D, Garg ML (2015). T2 relaxation time alterations underlying neurocognitive deficits in alcohol-use disorders (AUD) in an Indian population: A combined conventional ROI and voxel-based relaxometry analysis. Alcohol.

[27] Zhang G, Cheng Y, Liu B (2016) Abnormalities of voxel-based whole-brain functional connectivity patterns predict the progression of hepatic encephalopathy. Brain Imaging Behav doi:10.1007/s11682-016-9553-2 10.1007/s11682-016-9553-2 27138528

[28] Ferraro S, Grazzi L, Mandelli ML, Aquino D, Di Fiore D, Usai S (2012). Pain processing in medication overuse headache: a functional magnetic resonance imaging (fMRI) study. Pain Med.

[29] Lan CC, Tsai SJ, Huang CC, Wang YH, Chen TR, Yeh HL (2015). Functional connectivity density mapping of depressive symptoms and loneliness in Non-demented elderly male. Front Aging Neurosci.

[30] Radat F, Lanteri-Minet M (2011). Addictive behaviour in medication overuse headache: a review of recent data. Rev Neurol (Paris).

[31] Biagianti B, Grazzi L, Gambini O, Usai S, Muffatti R, Scarone S, Bussone G (2012). Decision-making deficit in chronic migraine patients with medication overuse. Neurol Sci.

[32] Ferraro S, Grazzi L, Muffatti R, Nava S, Ghielmetti F, Bertolino N (2012). In medication-overuse headache, fMRI shows long-lasting dysfunction in midbrain areas. Headache.

[33] Cauda F, Sacco K, Duca S, Cocito D, D’Agata F, Geminiani GC, Canavero S (2009). Altered resting state in diabetic neuropathic pain. PLoS ONE.

[34] Magis D, D’Ostilio K, Thibaut A, De Pasqua V, Gerard P, Hustinx R et al (2016) Cerebral metabolism before and after external trigeminal nerve stimulation in episodic migraine. Cephalalgia. doi:10.1177/0333102416656118 10.1177/0333102416656118 PMC5560481 27342225

[35] Tessitore A, Russo A, Esposito F, Giordano A, Taglialatela G, De Micco R (2011). Interictal cortical reorganization in episodic migraine without aura: an event-related fMRI study during parametric trigeminal nociceptive stimulation. Neurol Sci.

[36] Kim JH, Kim S, Suh SI, Koh SB, Park KW, Oh K (2010). Interictal metabolic changes in episodic migraine: a voxel-based FDG-PET study. Cephalalgia.

[37] Coppola G, Di Renzo A, Tinelli E, Iacovelli E, Lepre C, Di Lorenzo C (2015). Evidence for brain morphometric changes during the migraine cycle: a magnetic resonance-based morphometry study. Cephalalgia.

[38] Schwedt TJ, Chong CD, Wu T, Gaw N, Fu Y, Li J (2015). Accurate classification of chronic migraine via brain magnetic resonance imaging. Headache.

[39] Chen WT, Wang SJ, Fuh JL, Ko YC, Lee YC, Hamalainen MS, Lin YY (2012). Visual cortex excitability and plasticity associated with remission from chronic to episodic migraine. Cephalalgia.

[40] Vigano A, D’Elia TS, Sava SL, Auve M, De Pasqua V, Colosimo A (2013). Transcranial Direct Current Stimulation (tDCS) of the visual cortex: a proof-of-concept study based on interictal electrophysiological abnormalities in migraine. J Headache Pain.

[41] Schwedt TJ, Chong CD, Chiang CC, Baxter L, Schlaggar BL, Dodick DW (2014). Enhanced pain-induced activity of pain-processing regions in a case–control study of episodic migraine. Cephalalgia.

[42] Liu J, Lan L, Li G, Yan X, Nan J, Xiong S (2013). Migraine-related gray matter and white matter changes at a 1-year follow-up evaluation. J Pain.

[43] Fumal A, Laureys S, Di Clemente L, Boly M, Bohotin V, Vandenheede M (2006). Orbitofrontal cortex involvement in chronic analgesic-overuse headache evolving from episodic migraine. Brain.

